# Liquid biopsy by analysis of circulating myeloma cells and cell-free nucleic acids: a novel noninvasive approach of disease evaluation in multiple myeloma

**DOI:** 10.1186/s40364-023-00469-6

**Published:** 2023-03-08

**Authors:** Shuchan Li, Enfan Zhang, Zhen Cai

**Affiliations:** 1 Bone Marrow Transplantation Center, The First Affiliated Hospital, School of Medicine, Zhejiang University, No. 79, Qingchun Road, Hangzhou, Zhejiang China; 2 Institute of Hematology, Zhejiang University, Hangzhou, Zhejiang China

**Keywords:** Multiple myeloma, Liquid biopsy, Circulating tumor cell, Cell-free DNA, Cell-free RNA

## Abstract

Multiple myeloma (MM) is an incurable hematological cancer with high spatial- and temporal-heterogeneity. Invasive single-point bone marrow sampling cannot capture the tumor heterogeneity and is difficult to repeat for serial assessments. Liquid biopsy is a technique for identifying and analyzing circulating MM cells and cell products produced by tumors and released into the circulation, allowing for the minimally invasive and comprehensive detection of disease burden and molecular alterations in MM and monitoring treatment response and disease progression. Furthermore, liquid biopsy can provide complementary information to conventional detection approaches and improve their prognostic values. This article reviewed the technologies and applications of liquid biopsy in MM.

## Introduction

**Fig. 1:**
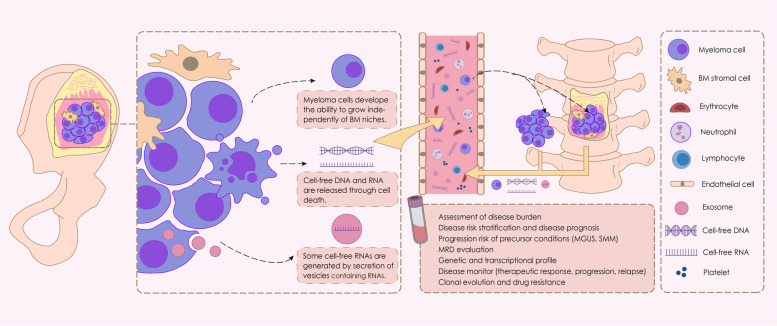
Schematic illustration of liquid biopsy in multiple myeloma (MM). We illustrated the role of circulating myeloma cells (CMMCs) in disease dissemination to distant bone marrow (BM) or extramedullary (EM) sites, and the utility of liquid biopsy (including CMMC and cell-free nucleic acids) in MM

Multiple myeloma (MM) is an incurable hematological cancer that is characterized by the abnormal proliferation of malignant plasma cells (PCs) in the bone marrow (BM). Currently, the diagnosis and evaluation of MM highly rely on BM sampling, which is invasive, painful, and difficult to repeat for serial assessments, highlighting the need for less invasive methods. The development of “liquid biopsies” opens up new avenues for noninvasive MM assessment and monitoring. Liquid biopsy is a diagnostic technique for identifying and analyzing circulating MM cells (CMMCs) and cell products produced by tumors and released into the peripheral blood (PB), particularly circulating cell-free nucleic acids (cf-NAs) (Fig. 1). Cell-free DNA (cfDNA) is primarily released into the circulation through cell death [1]. The first study on liquid biopsy in MM was published in 1993 and proposed that CMMCs were a measure of disease activity [2].

CMMCs or cf-NAs allow for the minimally invasive detection of disease burden and molecular alterations in MM and repeated sampling for disease monitoring. MM is characterized by intra-clonal heterogeneity and multifocal tumor deposition; occasionally, extramedullary (EM) lesions were inaccessible, limiting their molecule analysis. A single-site BM aspirate would be unrepresentative of disease infiltration and mutational profile. Liquid biopsy allows for the capture of comprehensive tumor heterogeneity. To date, the clinical use of liquid biopsy has been suggested for several malignancies, including hematological malignancies and solid tumors (e.g., breast and lung tumors). Patients with MM had greater cell-free tumor DNA (ctDNA) concentrations and CMMC counts per blood tube than those with advanced solid tumors [3], which supports the use of liquid biopsy in MM. This article provides a review of the technologies and applications of liquid biopsy in MM.

## Approaches for selection, enrichment, and isolation

The level of myeloma cells in the blood is much lower than that found in the BM [4, 5]. Next-generation flow cytometry (NGF)-based quantification detected only a median of approximately 1–1.9 CMMCs/uL in the PB from newly diagnosed multiple myeloma (NDMM) [6, 7]. The cfDNA level was low and variable in patients with MM (approximately 20.1–25.2 ng/mL of plasma) [3, 8–10]. cfDNA in the PB mainly originates from hematopoietic cells [11] and can be present in very low concentrations [12]. Different methods have been used to detect CMMCs and ctDNA with varying sensitivities and specificities (Table 1).

**Table 1:** The detection efficiency and sensitivity of different methods in liquid biopsy

Method	Detection efficiency and sensitivity	Reference
Wright–Giemsa-stained blood smears	CMMCs were detected in approximately 14.1%–20.8% of patients with NDMM at diagnosis	[13, 14]
Slide-based immunofluorescence	Sensitivity: 0.01% CMMCs were detected in 19.4%, 25%, and 80% of patients with MGUS, SMM, and NDMM, respectively	[15–18]
MFC (2-color: CD45 and CD38)	Sensitivity: 0.01% CMMCs were detected in 20%, 40%, 73%–83.6%, and 38.6% of patients with MGUS, SMM, NDMM at diagnosis, and MM before ASCT, respectively	[17, 19–22]
MFC (5-color: CD38, CD138, CD45, CD19, and CD56)	Sensitivity: 0.01% CMMCs were detected in approximately 69.2%–74.1%, 60.5%, 0%, and 14% of patients with NDMM at diagnosis, in PR, in CR, and at relapse, respectively	[23, 24]
MFC (6-color: CD38, CD138, CD45, CD19, cytoplasmic κ, and λ light chains)	Sensitivity: 20 cells/150,000 events (0.013%) CMMCs were detected in 24%, approximately 51.4%–67%, approximately 19.3%–19.4%, and 62/145 of patients with SMM, NDMM before therapy, MM before ASCT, and MM at relapse, respectively	[25–30]
MFC (7-color: CD38, CD138, CD45, CD19, CD56, cytoplasmic κ, and λ light chains)	Sensitivity: 0.01% CMMCs were detected in 60.1% and 18.8% of patients with NDMM at diagnosis and MM before ASCT, respectively	[31, 32]
2 tubes/MFC (7-color: CD38, CD138, CD45, CD19, CD56, cytoplasmic κ, and λ light chains)	Sensitivity: approximately 0.004%–0.0001% CMMCs were detected in 119/191 (approximately 67%) of patients with NDMM at diagnosis	[33, 34]
Magnetic cell sorting (MACS) (CD38 or CD138) combined with MFC (5-color: CD38, CD138, CD45, CD19, and CD56)	Sensitivity: 0.001% CMMCs were detected in 87.2%, approximately 83.7%–86%, approximately 5%–10%, and 85% of patients with NDMM at diagnosis, in PR, in CR, and at relapse, respectively	[23]
MACS (CD138) combined with MFC (6-color: CD38, CD138, CD45, CD19, CD56, and CD117)	CMMCs were detected in 55.5% and 28.6% of patients with MM with EM at diagnosis and NDMM without EM at diagnosis, respectively	[35]
MACS (CD138) combined with MFC (7-color: CD45, CD19, CD81, CD27, CD117, CD56, and CD200)	CMMCs were detected in 83.3% and 9.9% of patients with NDMM/MM at relapse and MM who achieved CR, respectively	[36]
NGF (2-tube/8-color)	Sensitivity: 0.0001% CMMCs were detected in approximately 92%–100%, 100%, 59%, 25%, 18%, 17%, and 100% of patients with NDMM at diagnosis, SMM, MGUS, macro focal MM, solitary plasmacytoma, MM who achieved CR/sCR, and relapsed/refractory multiple myeloma (RRMM), respectively	[6, 7, 37, 38]
CellSearch platform	CMMCs were detected in 98%, 93.7%, and approximately 56%–86% of patients with NDMM at baseline, intermediate/high-risk SMM, and MGUS, respectively	[39]
Epic platform	Sensitivity: one MM cell in 3*10^6^ WBCs	[40]
CD138-coated microfluidic device (Herringbone-shaped)	Sensitivity: < 10 CMMCs/mL using 1-mL sample	[41]
CD138-coated microfluidic device (Sinusoidal-shaped)	CMMCs were detected in 78% of patients with MGUS and 100% of those with SMM and MM	[42]
ASO-PCR of IGH rearrangements	Sensitivity: 0.001% CMMCs were detected in 13/16, 6/8, and 13/15 of patients with MGUS, SMM, and active MM, respectively	[4]
Real-time quantitative PCR of IGH rearrangements	Sensitivity: approximately 0.01%–0.001% CMMCs were detected in 67%, 43%, 25%, and 73% of patients with NDMM at diagnosis, NDMM before HDT for ASCT, NDMM 3 months after HDT, and RRMM at the time of relapse, respectively	[5, 43]
LymphoSIGHT assay of IGH and IGK rearrangements	Sensitivity: well below 0.0001% 1. CMMCs were detected in 78% of patients with MM using DNA assay and 96% of patients with MM using DNA and RNA assays 2. ctDNA was detected in 83% of patients with MM using DNA assay 3. Tumor clones were detected in 98% of patients with MM using the combination of CMMCs and ctDNA	[44]
Ion Torrent of IGH rearrangements	Sensitivity: 0.001% MM clones in cfDNA were detected in 100% of patients with MM at relapse	[12]
NGS of IGK and IGL rearrangements	MM clones in cfDNA were detected in 71.4% of patients with NDMM/MM at relapse and 22.2% of samples from MM who achieved CR. All ctDNA-detectable CR samples were from a patient with nonsecretory MM	[36]
NGS of IGH, IGK, and IGL rearrangements	CMMCs were detected in 71% of patients with MM at baseline. MM clones in cfDNA were detected in 100% of patients with MM at baseline. MM clones in CMMCs and/or cfDNA were detected in 91% and 41% of patients with MM with stable or progressive disease and MM with PR or better, respectively	[45]
ULP-WGS	Lower limit: TF ≥ 3% In NDMM/RRMM, ≥ 3% TF was detected in 76% cfDNA samples and 100% CMMC samples; ≥ 10% TF was detected in approximately 24%–32% cfDNA samples and in 31% CMMC samples In MGUS/SMM/NDMM/RRMM, ≥ 3% TF was detected in 58% cfDNA samples and 96% CMMC samples; ≥ 10% TF was detected in 17% cfDNA samples and 21% CMMC samples	[46–48]
LP-WGS	Lower limit: TF ≥ 5% ≥ 5% TF was detected in 62% of cfDNA samples from patients with RRMM, in 75% of cfDNA samples from patients with NDMM, and in none of cfDNA samples from patients with MM post-treatment	[49]

Wright–Giemsa-stained PB smear is the conventional method for identifying and counting PCs in the blood [13, 14, 50]. While this approach was simple and inexpensive, it could not identify cell clonality and was less sensitive than other methods. Slide-based immunofluorescence identified CMMCs using morphology and fluorescence staining [15, 16]. Single CMMC could further be isolated using fluorescence microscopy of CD138 + CD45 − cells, which was highly sensitive and specific although time- and labor-consuming [51]. The Epic Platform is an automated digital system that uses immunofluorescence to detect and characterize CMMCs based on morphological factors and levels of CD138 and CD45 expression. This test accurately identified all populations with MM CMMC with variable marker expression (positive or negative CD138) and could be further multiplexed with secondary biomarkers, including the phosphor-ribosomal protein S6 or CD56 [40, 52].

Multicolor flow cytometry (MFC)/fluorescence-activated cell sorting, alone or combined with magnetic cell sorting (MACS) enrichment (mainly with anti-CD138 antibody), is the most commonly used method for CMMC detection and isolation. The number of CMMCs detected by MFC correlated well with that detected by slide-based immunofluorescence microscopy [19]. However, there are some limitations. The sensitivity of MFC remained relatively low for the detection of extremely rare CMMCs, and a pre-enrichment step required sufficient starting CMMC concentrations. The heterogeneity of MFC instruments and detection markers resulted in variations in detection efficacy and the cut-off for CMMCs. The advent of NGF provides a possibility to adopt a standardized method for detecting CMMCs [7]. The purity of the NGF-sorted CMMCs has been confirmed by analysis of shared clonal mutations in BMPCs and CMMCs [37]. The CellSearch platform, which has received Food and Drug Administration approval for clinical use in several types of cancers, provided a more sensitive (when compared with MFC), highly reproducible, easily standardized, and high-throughput approach in CMMC detection. By combining with the DEPArray, the CellSearch–DEPArray system enabled the capture of a single CMMC [39].

Microfluidic devices were designed with microtraps whose sizes were tuned to physically capture a single CMMC with small volumes of samples and antibodies in a short time [53]. The device has high sensitivity (approximately < 10 CMMCs/mL of blood) [41] and meets the requirement for precise single-cell diagnostics using CMMCs. By combining the immunophenotypic and physical selections, anti-CD138 antibody-coated microfluidic channels were designed. The microfluidic-based CMMC counts and MFC analyses showed excellent correlation [41]. Another anti-CD138 antibody-coated microfluidic system permitted reversible cell capture. The antibody was attached via a linker, which could be degraded by enzymatic cleavage [42].

The nucleic acid-based method primarily detects CMMCs and ctDNA by identifying tumor-specific immunoglobulin (Ig) rearrangements or genetic abnormalities. The variable regions in Ig genes were transcribed in a patient-specific manner [35]. CMMCs and ctDNA from patients with MM had the same clonotypic Ig gene rearrangement as matched BM clonal PCs [12, 36, 54, 55]. Clonal Ig rearrangements were tracked using polymerase chain reaction (PCR)-based approaches, including quantitative PCR (qPCR) along with allele-specific oligonucleotides (ASOs) [4, 5, 8, 43], droplet digital PCR (ddPCR) [43], and next-generation sequencing (NGS) [12, 36]. A moderate agreement (approximately 80%) was observed between NGS of Ig rearrangement in cfDNA and MFC of CMMCs, indicating that cfDNA and CMMC analysis provided complementary information [36]. The main limitation of Ig rearrangement-based approaches was that they relied on the previously identified tumor clone.

Non-targeted approaches, including whole-genome sequencing (WGS), whole-exome sequencing (WES), and ultra-low pass WGS (ULP-WGS), allowed for genome-wide analysis. ULP-WGS (approximately 0.1 × coverage) provided a cost-effective approach for estimating genome-wide tumor fraction (TF) based on copy number aberration (CNA) profiles independent of prior knowledge of a patient’s tumor mutations [46]. However, other genetic aberrations (e.g., translocations) could not be assessed owing to the nature of the ULP-WGS analysis. These genome-wide analyses had lower assay sensitivity, which limits their use in patients with small TF (e.g., patients in the asymptomatic or pre-relapse stage) [56]. Deep-targeted sequencing approaches (e.g., NGS with a specific panel) have high sensitivity and can detect mutations in cfDNA that ULP-WGS or WES would miss [56]. One limitation of the targeted method is the requirement for prior identification of mutations in the primary tumor. A 14-gene cancer personalized profiling sequencing could detect all tumor PC-confirmed mutations in cfDNA when the variant allelic frequency (VAF) was ≥ 5% of mutations in BM tumor cells [56, 57]. Another 5-gene NGS panel that targeted all protein-coding exons of genes allowed for the detection of tumor-specific mutations in cfDNA at VAF as low as 0.25% (median 3.2%) [3]. The low DNA input hampered the utility of cfDNA with NGS in minimal residual disease (MRD) evaluation. A cross-platform evaluation of NGS-based ctDNA assays showed that, when the VAF was more than 0.5%, ctDNAs were detected with high sensitivity, precision, and reproducibility by all methods [58]. Generally, ddPCR and qPCR, as well as ddPCR and an NGS-based approach, demonstrated excellent correlation in mutation identification [10, 43]. However, in some studies, ddPCR was more sensitive (can detect mutation frequencies as low as 0.005%) than NGS and identified some mutations in cfDNA missed by NGS [10, 59–61].

## Mechanisms explaining multiple myeloma trafficking through peripheral blood disease dissemination

It was hypothesized that as the disease progressed, myeloma cells developed the ability to grow independently of BM niches, translocate into the blood, and re-home at distant sites in the BM and other tissues. The mechanisms underlying the migration of PCs from the BM to the circulation and EM spread through PB dissemination remained unclear. Although, in general, CMMCs displayed overlapping immunophenotypic [7, 35, 62, 63], genomic [37], and transcriptomic [64] profiles with BM tumor PCs, there could be minor but consistent differences between myeloma cells in the PB and BM that could indicate hallmarks associated with cell translocation and disease dissemination.

A more immature and less proliferative immunophenotype was displayed on CMMCs. CMMCs expressed significantly lower levels of CD28, CD38, CD138, CD81, CD27, CD52, CD117, Vs38c, and Ki67 [7, 36, 62–65]. Virtually all CMMCs were in the sub-G0/G1 phase of the cell cycle [62], and the gene expression (e.g., CENPF or CDC6) and pathways (DNA repair, mitotic spindle formation, and G2M checkpoint) involved in the cell cycle were significantly downregulated in CMMCs [65]. Furthermore, CMMCs displayed lower expression of integrin and adhesion molecules, including CD11a, CD11c, CD29, CD33, CD49d, CD49e, [62] and CD56 [7, 35, 42, 52, 62, 63], which potentially enhanced its capacity to exit into the PB. Sphingosine 1-phosphate receptor 2 (S1RP2), whose inhibition significantly promoted cell migration and invasion via NF-kB pathway phosphorylation, was expressed at a significantly lower level in CMMCs [66]. The expression of adhesion-related genes (CD44 and galectin 1) and the pathway involved in epithelial–mesenchymal transition (EMT) were significantly upregulated in CMMCs. CD44 knockdown impaired cell migration and adhesion to fibronectin, whereas EMT is a significant process in tumor metastasis [65]. Furthermore, compared with BM clonal PCs, CMMCs demonstrated greater clonogenic potential in the colony and cluster formation in vitro and exhibited a circadian distribution by actively migrating to PB and metastasizing to other sites during the patients’ resting period [62].

It is unclear whether myeloma cells with distinct genetic features are more prone to spread the disease. Some data indicated that the CMMC population represented a more genetically abnormal subclone than the BM clonal PC or CMMC population from the early disease stage and that an appreciable number of mutations were identified in EM clones although absent in BM clones were identified in CMMC [37, 46, 51]. By comparing the degree of genomic similarity between BMPC, CMMC, and PC from EM, it can be determined that CMMCs are the most likely precursor of EM plasmacytomas and may act as a cellular bridge between BM and EM lesions [67]. Another hypothesis suggested that the spread of MM was driven by differential gene expression rather than unique genetic alterations. Some studies found that CMMCs had considerably increased levels of altered genes and pathways associated with hypoxia, inflammation, tumor migration, invasiveness, and metastasis, suggesting that the hypoxic and inflammatory microenvironment in BM niches would inhibit myeloma cell proliferation, forcing their migration into the PB and invasion of other niches [65]. Another possible mechanism is increased auto-secretion and self-feeding of myeloma cells. Chemokine CXCL12, which is normally expressed in BM stromal cells and is involved in CXCR4-dependent BM retention, was found to be significantly upregulated in MM CMMCs, suggesting that CMMCs generated a self-feed loop and released themselves from BM retention, thereby promoting egress to the PB [35].

## Disease burden assessment

Sequential liquid biopsy examinations may provide a noninvasive real-time measure of tumor burden and a more comprehensive quantification of whole-body tumor burden than single-site BM biopsy examinations. The detection rate, the absolute number of CMMCs [7, 23, 39, 42, 68, 69], and the TF in CMMCs [46] were correlated with the disease status, which progressed from solitary plasmacytoma to monoclonal gammopathy of unknown significance (MGUS), smoldering multiple myeloma (SMM), and NDMM/MM at relapse. CMMCs were more frequently found in patients with active-relapsing MM than in those with stable disease (SD) [25]. The absolute number of CMMCs was significantly higher at baseline and relapse than that in MM undergoing treatment, and further decreased correlating to the depth of response, that is, partial response (PR), very good partial response (VGPR), and complete response (CR) [23, 26, 39, 41, 42]. The presence of CMMCs, the absolute number of CMMCs, and the TF in CMMCs were all significant predictors of clinical scores or indicators of high disease burden, including advanced Mayo Clinic Index and Spanish criteria of MGUS [7], high risk and ultra-high risk SMM [39], advanced Durie–Salmon (DS) stage [13, 23, 33], International Staging System (ISS) stage [6, 13, 23, 27, 33, 34, 39, 63, 70, 71] and Revised-ISS (R-ISS) stage [7, 33, 34, 46, 70], higher serum levels of beta2-microglobulin (β2-MG) [13, 20, 23, 25, 33, 63, 70] and lactate dehydrogenase (LDH) [20, 25, 26, 28, 33, 34, 70, 71], lower serum level of albumin [70], lower hemoglobin [13, 23, 24, 33, 63, 70] and platelet counts [14, 24], higher serum creatinine (Scr) [23, 25, 33], and advanced bone destruction [23, 33]. Chromosomal abnormalities (CAs) play a significant role for predicting the risk of patients with MM. CMMC levels were correlated with a higher incidence of high-risk cytogenetic abnormalities [6, 13, 20, 27, 28, 34, 63, 70–72], a lower incidence of hyperdiploidy [26], and standard-risk cytogenetic abnormalities [34]. CMMC abundance was associated with disease burden in the BM, including the tumor cell involvement [6, 7, 13, 14, 20, 23–25, 28, 33, 34, 39, 63, 70, 71] and the myeloma clone levels of Ig rearrangements in the BM [44]. The correlation between the percentage of tumor cells in the PB and BM adjusted better to a nonlinear rather than a linear trend [6]. The cancer cell fraction (CCF) of clonal mutations in CMMCs was only modestly correlated with the CCF of clonal mutations in myeloma cells in the BM owing to the presence of mutations that were clonal in one compartment but subclonal in another [37].

The cfDNA concentrations and the TF in cfDNA were correlated with the disease status and revealed significant differences between patients with MGUS, SMM, NDMM/MM at relapse, and post-treatment MM [3, 46, 49, 56, 57]. A previous study found that the TF in cfDNA from MM was 4.5 times higher than that in cfDNA from MGUS and SMM [56]. The cfDNA levels were observed to be significant predictors of clinical scores or markers of high disease burden, including advanced ISS stage [9, 57, 73] and R-ISS stage [46, 73], elevated levels of LDH [3, 9, 47, 73] and β2-MG in serum [9], more EM disease in positron emission tomography-computed tomography (PET-CT) [47, 74], or osteolytic lesions [48, 74]. Most patients showed a positive correlation between the frequencies and VAF of mutations [57, 73, 75, 76], the TF based on CNAs [3, 49, 74, 76], and the frequencies of MM clones (Ig rearrangements) [36] in paired myeloma cells in the BM and cfDNA. However, the ctDNA level only showed a conditional correlation with myeloma cell infiltration in the BM. Although some studies found that patients with a high ctDNA level had more BM infiltrations [47, 48, 57, 73], no quantificational correlation was found between the VAF of tumor-related mutations in cfDNA and BM MM cell infiltration [10], which could be explained by BM heterogeneity and the presence of EM lesions. According to a previous report, patients with short progression-free survival (PFS) and high tumor burden by cfDNA were observed to have inconsistently low BM infiltration. This suggests that cfDNA is less prone to spatial and technical bias than a BM biopsy and can assess a more thorough disease burden than a single-site BM biopsy [47].

There have been few studies that directly compare the disease burden mirrored by CMMCs and cfDNA. Patients with higher molecular tumor burden index levels in ctDNA had higher percentages of CPCs [73]. A comparison of the frequency of MM clones by IGK or IGL rearrangement in cfDNA by NGS and CMMC levels by MFC revealed 80% concordance, and the cell-based approach achieved greater patient coverage than the NGS assay [36]. Another study found a 30% discordance in the frequencies of MM clones by IGH and light chain (LC) rearrangement in cfDNA and CMMCs, indicating that cfDNA may not be entirely generated by CMMCs and may reflect overall tumor burden [45]. Studies focusing on the TF evaluated using ULP-WGS found that the TF in CMMCs was higher than that in paired cfDNA. Moreover, they showed a significant difference in the TF from matched cfDNA and CMMCs in a specific individual, suggesting that analyzing both cfDNA and CMMCs may broaden the applicability of liquid biopsies [46, 56].

## Utility in risk stratification and disease prognosis

Several studies have confirmed the CMMC level at diagnosis, after treatment, and at remission before/after autologous stem cell transplantation (ASCT) as a prognostic factor for therapeutic response and progression (or early relapse) in MGUS, SMM, and MM, independent of several known risk factors, including ISS/R-ISS stage and high-risk cytogenetics (Table 2). In 2005, it was first proposed that CMMCs had a prognostic value, independent of age, albumin, and β2-MG [19]. When CMMCs were modeled as a continuous predictor, the risk of progression and relapse continuously increased in patients with MM with undetectable CMMCs and those with increasing CMMC percentages [6]. However, the cut-off that separated patients with different prognosis in several trials using various quantitative approaches was different, thereby limiting their clinical utilization. Ravi et al. and Granell et al. observed that survival was similar between NDMM with 5%–19% and ≥ 20% CMMCs measured on a blood smear stained with Wright–Giemsa, which was significantly poorer than those with < 5% CMMCs. Those with ≥ 5% CMMCs had significantly poorer survival than those with standard-risk cytogenetics MM and high-risk MM [14, 50]. Based on these two studies, the International Myeloma Working Group (IMWG) revised the definition of plasma cell leukemia (PCL) to include the presence of 5% or more CMMCs in blood smears [77]. Moreover, the dynamic of CMMCs at different time points showed a great prognostic value. Patients with undetectable CMMCs at the last follow-up in sequential monitoring showed better outcomes than those with CMMCs at the last follow-up [38]. By evaluating the CMMC status at diagnosis and before ASCT, undetectable CMMCs at both time points were a biomarker predicting a high rate of post-transplant stringent CR. The presence of CMMCs following induction therapy was a factor in inferior survival [26, 72], and this adverse impact was not overcome by maintenance therapy [26, 31]. Regarding cfDNA, the level of the tumor-associated IGH sequence (≥ 4.7% of total reads) in cfDNA before therapy was a prognostic factor for inferior PFS [12]. The high ctDNA level (≥ 10% TF in cfDNA) at screening and after two cycles of treatment (C3D1) was an independent factor for inferior PFS [47]. Furthermore, the high cfDNA concentration (> 25.2 ng/mL of plasma) was an independent factor for inferior PFS and overall survival (OS) [9]. The numbers and VAF of driver genes in cfDNA were independent factors for inferior OS, and its changes after treatment (C1D5) were associated with PFS [10, 78].

**Table 2:** The role of liquid biopsy in predicting therapeutic responses and disease prognosis in MM and precursor conditions

Sample	Detection time	Method	Cut-off	Prognostic value	Reference
NDMM	At diagnosis	Wright–Giemsa-stained PB smears	≥ 2% CMMCs per 100 nucleated cells on PB smears	1. A prognostic factor for inferior PFS and OS (not independent) 2. The PFS and OS of MM with CMMCs were comparable with primary PCL	[13]
NDMM	At diagnosis	Wright–Giemsa-stained PB smears	≥ 5% CMMCs per 100 nucleated cells on PB smears	A prognostic factor for inferior OS independent of age, Scr, DS stage, and ISS stage	[14, 50, 77]
MGUS	/	Slide-based immunofluorescence	Presence of CMMC	1. An independent prognostic factor for inferior PFS and OS 2. Patients with CMMCs were twice as likely to progress than those without CMMCs	[15]
SMM	/	Slide-based immunofluorescence	CMMCs > 5,000 × 10^6^/L and/or > 5% cytoplasmic Ig-positive PCs	1. An independent prognostic factor for inferior TTP and OS 2. A prognostic factor for higher incidences of 2- and 3-year progression	[16]
NDMM	At diagnosis	Slide-based immunofluorescence	≥ 4% cytoplasmic Ig-positive CMMCs	An independent prognostic factor for inferior OS	[17]
NDMM	At diagnosis	MFC (2-color)	Presence of CMMC	An independent prognostic factor for inferior PFS and OS	[22]
NDMM	At diagnosis	MFC (2-color)	> 10 CMMCs/50,000 events	First demonstration of its independent prognostic value of inferior OS	[19]
NDMM	At diagnosis	MFC (2-color)	≥ 41 CMMCs/50,000 events	A prognostic factor for inferior PFS and OS independent of standard-risk cytogenetics	[21]
NDMM	Before ASCT	MFC (2-color)	Presence of CMMC	A prognostic factor for inferior TTP (early relapse after ASCT) and OS independent of cytogenetics and response status after induction therapy	[20]
NDMM	At diagnosis	MFC (5-color)	CMMC ≥ 0.02%	Independent prognostic factor of inferior PFS and OS	[24]
SMM	/	MFC (6-color)	≥ 150 CMMCs/150,000 events	1. Independent prognostic factor for inferior TTP and OS 2. A prognostic factor for higher incidence of 2-year progression	[30]
NDMM	At diagnosis	MFC (6-color)	Presence of CMMC and ≥ 400 CMMCs/150,000 events	Prognostic factors for inferior TTNT and OS independent of cytogenetic status	[28, 29]
MM in a plateau, RRMM	After therapy	MFC (6-color)	Presence of CMMC and ≥ 100 CMMCs/150,000 events	1. MM in a plateau with CMMCs had inferior OS (independent) 2. RRMM with ≥ 100 CMMCs/150,000 events had inferior OS (independent)	[25]
NDMM	Before ASCT	MFC (6-color)	Presence of CMMC	1. A prognostic factor for PFS and OS independent of post-transplant sCR 2. A prognostic factor for post-transplant response status	[27]
NDMM	At diagnosis, before ASCT and day 100 post-transplant	MFC (6-color)	1. Presence of CMMC 2. Dynamics of CMMCs at diagnosis and before ASCT (− / −), (+ / −), (+ / +), (− / +)	1. CMMC (+ / +) or (− / +) were factors for lower incidence of pretransplant ≥ VGPR and post-transplant sCR 2. CMMC (+ / +) or (− / +) was an independent factor for inferior PFS and OS 3. Patients with CMMCs at day 100 post-transplant had inferior PFS and OS	[26]
MM with EM	/	Combination of MACS and MFC (6-color)	Presence of CMMC	The presence of CMMCs in patients with EM disease had worse OS	[35]
NDMM	At diagnosis	MFC (7-color)	≥ 0.10% CMMCs/150,000 events	A prognostic factor for inferior PFS and OS independent of R-ISS stage and age	[32]
NDMM	At diagnosis	MFC (2-tube/7-color)	≥ 0.038% CMMCs	1. An independent prognostic factor for inferior PFS and OS 2. A factor for higher incidence of ≥ VGPR and ≥ PR	[33]
Transplant-eligible NDMM	At diagnosis	MFC (2-tube/7-color)	≥ 0.07% CMMCs (≥ 5 cells/μL)	1. A factor for lower incidences of MRD negativity and ≥ CR at premaintenance 2. A factor for inferior PFS and OS independent of ISS, cytogenetics, and LDH level 3. A similar prognostic value between the cut-off value and continuous variable	[34]
NDMM	Before ASCT	MFC (7-color)	Presence of CMMCs	1. A factor for lower incidence of VGPR or better 2. A prognostic factor for inferior PFS, independent of ISS stage, cytogenetics, and maintenance therapy 3. The presence of CMMC enhanced the stratification of VGPR or better	[31]
MGUS, SMM, MM	At diagnosis	MFC (8-color)	> 0.0035% CMMCs	An independent prognostic factor of inferior PFS and OS	[68]
MGUS, SMM, MM	At diagnosis	NGF	≥ 0.058 CMMCs/µL (for MGUS) ≥ 0.1 CMMCs/μL (for SMM and MM)	1. A factor for MGUS of higher incidence of progression in 30 months 2. A factor for SMM of higher incidence of progression to MM in 2 years 3. A factor for MM of inferior PFS and OS independent of CR status or MRD status	[7]
Treated MM	After therapy	NGF	1. Presence of CMMC 2. Kinetics of CMMCs	1. An independent prognostic factor for inferior PFS 2. The presence of CMMC enhanced the stratification of CR/sCR 3. Patients with CMMC − / − or + / − in sequential monitoring showed better PFS than those with CMMC + / + or − / + independent of sIF status	[38]
NDMM	At diagnosis	NGF	≥ 0.01% CMMCs (0.6 CMMCs/mL)	1. A factor for inferior PFS independent of ISS stage, LDH, and cytogenetics 2. A prognostic factor for inferior PFS independent of CR status and MRD status	[6]
NDMM	At remission	CellSearch platform	≥ 100 CMMCs/4 mL of blood	A prognostic factor for inferior PFS and OS	[39]
NDMM	At diagnosis and 3 months after HDT for ASCT	ASO-qPCR of IgH rearrangement	Presence of CMMC	1. At diagnosis: a prognostic factor for inferior EFS 2. Three months after HDT for ASCT: a prognostic factor for inferior EFS and OS	[5]
RRMM	Before therapy and in remission	NGS (Ion Torrent) of IgH rearrangement	≥ 4.7% of total reads (before therapy) 10^−5^ or 10^−4^ of total reads (at remission)	1. ctDNA levels before therapy were a prognostic factor for inferior PFS 2. ctDNA levels at remission were a prognostic factor for inferior PFS	[12]
NDMM	At diagnosis	ASO-qPCR of IgH rearrangement	Positive + PNQ > 50	A prognostic factor for lower CR rates	[8]
RRMM	At screening and after two cycles of treatment	LP-WGS	≥ 10% TF	1. ctDNA levels at screening were a prognostic factor for inferior PFS 2. ctDNA levels at C3D1 were an independent factor for inferior PFS 3. ctDNA levels at C3D1 enhanced the stratification of SD and PR	[47]
MM	/	/	cfDNA > 25.2 ng/mL plasma	ctDNA levels were a prognostic factor for inferior PFS and OS	[9]
NDMM	/	ddPCR (BRAF, KRAS, and NRAS)	Presence of mutations VAF > 5% trimmed mean value	The presence of mutations and ctDNA levels were related to inferior OS	[79]
NDMM, RRMM	At screening and on C1D5	OMD and ddPCR	1) ≥ 2 plasma-specific mutations or > 1% FA 2) Presence of TP53 mutation 3) FA of ctDNA decrease on C1D5	1. OS was significantly inferior in MM with a high level of mutations in cfDNA 2. OS was significantly inferior in MM with TP53 mutation in plasma 3. Median PFS: significantly inferior in MM with no change or even increasing ctDNA levels at C1D5	[78, 80]

The CMMC assay defined high-risk disease independently of cytogenetics by fluorescence in situ hybridization (FISH) and ISS, and its quantification improved the stratification of these traditional parameters. Several prognostic models that combine conventional variables/scoring systems and perform well in prognostic stratification have been developed (Table 3). For example, a nomogram that included CMMC, Scr, and LDH showed better risk-stratifying ability than the DS stage, ISS, and R-ISS stage [33]. The CMMC level at diagnosis in NDMM was observed to increase the stratification of cases with standard-risk cytogenetic changes [21, 29]. The presence of CMMCs before ASCT increased the stratification of high-risk cytogenetic changes [20, 27]. Furthermore, to predict prognosis independent of the R-ISS stage, age, and high-risk cytogenetics and improve the risk-stratifying ability of the R-ISS stage or cytogenetics, Abe et al. developed a PET-CMMC staging system that combined CMMC and imaging characteristics from PET-CT (presence of more than three focal lesions with or without EM disease) [32]. Based on the genetic profile of ctDNA in MM, a three-factor nomogram (age ≥ 65 years, DNA repair pathway mutation, and/or transcriptional regulation pathway mutation in ctDNA) was constructed to predict the PFS of patients with NDMM [73].

**Table 3:** Prognostic models combining liquid biopsy with other conventional parameters

Sample	Method	Model	Reference
MGUS	Slide-based immunofluorescence	Prognostic score for PFS: 0–3 1. Presence of CMMCs 2. M protein ≥ 2 g/L in the PB 3. Disease type: non-IgG heavy chain	[15]
SMM	Slide-based immunofluorescence	Prognostic score for TTP: 0–2 1. CMMCs > 5,000 × 10^6^/L and/or > 5% cytoplasmic Ig-positive PCs 2. M protein spike ≥ 3 g/dL in the PB	[16]
NDMM	Slide-based immunofluorescence	Prognostic score for OS: 1. CMMC ≥ 4% 2. the BMPC labeling index (LI) ≥ 1%	[17]
NDMM	MFC (2-color)	Prognostic score (CMMC + ISS stage) for OS: 0–3 1. > 10 CMMCs/50,000 events at diagnosis 2. β2-microglobulin > 3.5 mg/L 3. Albumin < 3.5 g/dL	[19]
NDMM	MFC (2-color)	For PFS and OS: ≥ 41 CMMCs/50,000 events at diagnosis increased the stratification of NDMM with standard-risk cytogenetics but not of NDMM with high-risk cytogenetics	[21]
NDMM	MFC (2-color)	Prognostic score for PFS and OS: 1. Presence of CMMCs 2. R-ISS stage (R-ISSII)	[22]
MM with ASCT	MFC (2-color)	Prognostic score for PFS and OS: 0–2 1. Presence of CMMCs before ASCT 2. High-risk cytogenetics	[20]
MM with ASCT	MFC (6-color)	Prognostic score for OS: 1. Presence of CMMCs before ASCT 2. High-risk cytogenetics	[27]
NDMM	MFC (6-color)	Prognostic score for TTNT and OS: 1. ≥ 400 CMMCs/150,000 events (≥ 5 CMMCs/μL) at diagnosis 2. R-ISS stage (R-ISSII)	[28]
MM	MACS (CD138) combined with MFC (6-color)	Prognostic score for OS: 1. Presence of CMMCs 2. Presence of EM lesions	[35]
NDMM	MFC (7-color)	The PET-CMMC staging system for PFS and OS: 1. CMMCs ≥ 0.10% of the total mononuclear cells at diagnosis 2. Presence of > 3 focal lesions with or without EM disease in PET-CT The PET-CMMC system combined with the R-ISS stage The PET-CMMC system combined with high-risk cytogenetics	[32]
NDMM	MFC (7-color)	Nomogram for PFS and OS: 1. ≥ 0.038% CMMCs at diagnosis 2. Creatine and LDH levels	[33]
NDMM	MFC (7-color)	Prognostic score (CMMC + R-ISS) for PFS and OS: 1. ≥ 0.07% CMMCs (≥ 5 cells/μL) at diagnosis 2. R-ISS stage (R-ISSII) Prognostic score (CMMC + MRD) for PFS and OS: 1. ≥ 0.07% CMMCs (≥ 5 cells/μL) at diagnosis 2. Premaintenance MRD status	[34, 71]
NDMM	MFC (8-color)	Prognostic score (CMMC + R-ISS) for PFS and OS: 1. ≥ 0.105% CMMCs at diagnosis 2. R-ISS stage (R-ISSIII)	[72]
MGUS, SMM, MM	MFC (10-color)	Risk stratification for PFS and OS: 1. ≥ 0.0035% CMMCs of total leukocytes at diagnosis 2. High LDH and/or β2-microglobulin > 5.5 mg/L 1. High CMMCs combined with Mayo risk stratification in MGUS 2. High CMMCs combined with IMWG risk stratification in SMM 3. High CMMCs combined with R-ISS in MM	[68]
NDMM	NGF	Prognostic score for PFS: 1. ≥ 0.1 CMMCs/μL of blood at diagnosis 2. sIF status (≥ VGPR or not) Prognostic score for PFS: 1. ≥ 0.1 CMMCs/μL of blood at diagnosis 2. MRD status in the BM evaluated by NGF	[7]
NDMM	NGF	Prognostic score for PFS in all MM cohorts: 0–2 1. Presence of CMMCs after therapy 2. sIF status Prognostic score for PFS in MM achieved CR/sCR: 0–2 1. Presence of CMMCs after therapy 2. MRD status in the BM evaluated by NGF Prognostic score for PFS in longitudinal monitoring: 1. Changes of sIF status (− / − , − / + , + / + , + / −) 2. Changes of CMMC status (− / − , − / + , + / + , + / −)	[38]
NDMM	NGF	Prognostic score (CMMC + R-ISS) for PFS and OS: 0–4 1. ≥ 0.01% CMMCs (0.6 CMMCs/mL) at diagnosis 2. R-ISS stage (three factors)	[6]
RRMM	ULP-WGS	Stratification (cfDNA + IMWG response criteria) for PFS: 1. ≥ 10% TF in cfDNA after two cycles of treatment 2. IMWG response status (SD or PR)	[47]
SMM	/	Stratification (cfDNA + GEP70) for PFS and OS: 1. cfDNA > 25.2 ng/mL of plasma 2. GEP70 (high-risk or low-risk)	[9]
NDMM	NGS	Nomogram for PFS: 1. Age ≥ 65 years 2. DNA repair pathway mutation in ctDNA 3. Transcriptional regulation pathway mutation in ctDNA	[73]
MGUS, MM	RNA-seq	Ten-gene model in cf-mRNA: distinguish MM from MGUS and MGUS from non-cancer cases AIDA, CA1, EPB42, HBG1, HBG2, CENPE, CPOX, and NUSAP1, NEK2, ELL2	[81]

The ISS is the most reliable staging system in MM, and CA was integrated into the R-ISS. However, great heterogeneity in clinical characteristics and outcomes was observed in cases within identical R-ISS risk groups, particularly in the R-ISS II group [82–84], indicating the need for new parameters. Several studies presented in Table 3 have confirmed that the presence and quantification of CMMCs further improved the risk stratification of patients with different prognosis in the identical ISS and R-ISS stages [6, 19, 22, 28, 34, 68, 71]. A previous study defined an ultra-high-risk group by combining R-ISS stage III and CMMC ≥ 0.105% at diagnosis. They observed a trend for better survival in patients in the R-ISS III stage with CMMC < 0.105% than those in the R-ISS II stage and even those in the R-ISS I stage with a high level of CMMCs [72]. Deshpande et al. observed that gene expression profiling 70-gene (GEP70) high-risk patients had significantly higher cfDNA concentrations and TF in ctDNA with ULP-WGS than low-risk patients [9].

Furthermore, CMMC and cfDNA quantification could predict prognostic risk regardless of BM MRD and serum immunofixation electrophoresis (sIF) status [7, 20, 27] and discriminate between patients with different prognosis despite identical IMWG response depth or BM MRD status. The presence of CMMC at diagnosis further discriminated patients with inferior PFS in patients with identical post-treatment status (both in the ≥ VGPR and < VGPR groups) [7]. The presence of CMMC before ASCT discriminated patients with inferior PFS in patients who achieved VGPR or better [31]. Another scoring system that combined the CMMC status after treatment and the sIF status divided patients into three groups. Patients with persistent negative CMMC had the best prognosis regardless of their sIF status, whereas persistent positive CMMC was a predictor for inferior prognosis even in patients with persistent negative sIF [38]. In patients who achieved CR, those with > 10^−4^ tumor-associated Ig rearrangement in cfDNA showed the worst PFS [12]. In patients with PR or SD, those with ≥ 10% TF in cfDNA by ULP-WGS after two cycles of treatment showed inferior PFS [47]. A prognostic model combining the CMMC level at diagnosis and the BM MRD status at premaintenance showed the best prognosis in the CMMC − /MRD − group [34, 71]. Furthermore, the CMMC + /MRD − group had a better prognosis than the CMMC − /MRD + group, implying that BM MRD negativity could partially revoke the adverse effect of a high CMMC level [34]. Furthermore, other studies found that the presence of CMMC at diagnosis and after treatment further distinguished patients with poor PFS regardless of the BM MRD status [7, 38]. Only attaining MRD negativity (rather than CR) resulted in a statistically significant increase in PFS [6].

## Risk stratification of precursor conditions

MGUS and SMM are heterogeneous precursor states of MM. The rates of transformation from MGUS and SMM to active MM are approximately 1% and 10% annually, respectively [85]. A previous study identified the following two distinct entities of patients with MGUS: a group of patients destined to progress and another group remaining in a stable condition for a long time [86]. Identification of patients with a high risk of progression and detection of the progression at early stages would allow earlier intervention and improve the outcome. The noninvasive nature of liquid biopsy made it feasible in the routine screening of MM transformation. Compared with those without CMMCs, it was observed that patients with MGUS with CMMCs were twice as likely to experience progression to a more aggressive PC disease. A model predicting the progression risk of MGUS was constructed by combining the CMMC status, the type of heavy chain, and the level of monoclonal protein (M protein) [15]. Compared with those with no risk factors, the risk of progression in 2–3 years was 2.2 times higher in patients with SMM with ≥ 5,000 × 10^6^/L CMMCs or M protein level of ≥ 3 g/dL and 14 times higher in those with SMM with high M protein and CMMC levels [16]. Gonsalves et al. found ≥ 150 CMMCs as the biomarker of SMM for predicting 2-year progression with 97% specificity and 78% positive predictive value (PPV), which was better than the Mayo Clinic risk model [30]. Vasco-Mogorrón et al. observed that CMMC > 0.0035% was an independent adverse factor for PFS and OS in MGUS and SMM. By combining the level of CMMC, β2-MG, and LDH in serum, they constructed a prognostic model for MGUS and SMM and found that the annual progress rate was three times lower in low-risk MGUS patients with CMMCs < 0.0035% and 10 times higher in high-risk patients with CMMCs > 0.0035% than the average annual progress rate (approximately 1%) in MGUS [68]. Sanoja-Flores et al. observed that significantly higher rates of MGUS with ≥ 0.058 CMMC/µL progress to SMM and MM at 30 months, and SMM with ≥ 0.1 CMMC/μL progress to MM at approximately 2 years [7]. Foulk et al. reported that the CMMC level at baseline was a good predictor of disease progression of MM, corresponding to M protein, BMPCs, and the serum free light chain (sFLC) ratio [39]. By analyzing the cell-free messenger RNA (cf-mRNA) using RNA sequencing (RNA-seq), a selected cf-mRNA panel recapitulated the transition from MGUS to MM and distinguished normal controls and patients with MGUS from those with MM [81].

## Minimal residual disease evaluation

MRD evaluation has been accepted as a sufficient endpoint in disease assessment in MM, whose presence was considered as the source of recurrence for MM, and BM examination was the best indicator for detecting MRD. MRD evaluation in MM has been evaluated using MFC, NGS, and the NG of BM samples or image evaluation using PET-CT [87]. Although the utility of liquid biopsy (CMMC and ctDNA) in MRD evaluation has been confirmed in multiple types of solid tumors (e.g., tumors in the breast, prostate, bladder, colorectum, or lungs) [88], whether the persistence of CMMC/ctDNA in patients with MM could be a surrogate of BM MRD positivity remained unknown. In a study with a small sample size by Biancon et al., MRD evaluated by MFC with BM samples showed complete concordance with ctDNA analysis by NGS of IGH rearrangements [12]. However, in most studies shown in Table 4, undetectable CMMC/ctDNA has been observed in a significant proportion of patients with positive BM MRD (low negative predictive value), whereas MRD in the PB is constantly positive in patients with positive BM MRD (high PPV). These observations suggested that negative MRD in the PB may still not serve as a sufficient surrogate for negative BM MRD in MM, whereas persistent positive PB MRD may reflect the positive BM MRD and avoid invasive BM evaluation. According to *International harmonization in performing and reporting minimal residual disease assessment in multiple myeloma trials* proposed in 2021, although MRD evaluation in the PB is convenient and may overcome limitations of patchy BM involvement or EM disease, further investigation and cross-validation using BM-based MRD assays are required to achieve similar sensitivity with BM MRD evaluation [89].

**Table 4:** Comparison of MRD evaluation in the BM and PB

Sample	Method	Result	Reference
CMMC n = 122	**MRD in BM**: 5-color MFC **MRD in PB**: MACS (CD138) combined with 5-color MFC	1. MRD-positive BM samples were accompanied by PB-MRD-positive results in 88% of corresponding PB samples 2. 100% of MRD-negative BM samples were accompanied by MRD-negative PB samples in NDMM, RRMM and MM achieved PR	[23]
CMMC n = 45	**MRD in BM and PB**: 8-color MFC	1. 100% of PB-MRD-positive patients were BM-MRD-positive 2. 56% of PB-MRD-negative patients were BM-MRD-negative	[72]
CMMC n = 137	**MRD in BM and PB**: NGF	1. 100% of PB-MRD-positive patients were BM-MRD-positive 2. 46/101 of PB-MRD-negative patients were BM-MRD-negative	[38]
CMMC n = 42	**MRD in BM and PB**: RT-qPCR of IGH rearrangements	1. 100% of BM-MRD-negative patients were PB-MRD-negative before/after transplantation 2. 47% of BM-MRD-positive patients were PB-MRD-positive before transplantation 3. 33% of BM-MRD-positive patients were PB-MRD-positive after transplantation	[5]
cfDNA n = 42	**MRD in BM and PB**: NGS of clonal Ig gene rearrangements	1. 89% of PB-MRD-positive patients were BM-MRD-positive 2. 36% of PB-MRD-negative patients were BM-MRD-negative	[55]
cfDNA n = 22	**MRD in BM**: MACS (CD138) combined with 8-color MFC **MRD in PB**: NGS of IGH rearrangements	The BM-MRD status evaluated by MFC was highly correlated with the PB-MRD evaluated by ctDNA analysis	[12]
cfDNA n = 45	**MRD in BM**: 8-color MFC **MRD in PB**: ASO-qPCR of IGH rearrangements	1. 5/6 of BM-MRD-negative patients were PB-MRD-negative 2. 2/6 of BM-MRD-positive patients were PB-MRD-negative	[8]

## Genetic and transcriptional profile identification

Spatial genomic heterogeneity in MM has been confirmed by multi-region sequencing in BM samples and even at different EM lesion sites [90, 91]. Liquid biopsies could offer a more thorough clonal heterogeneity profile in MM. It would be helpful to sequence matched CMMCs, cf-NAs, and BM and EM samples from patients with MM to validate the use of liquid biopsies in noninvasive molecular screening (Table 5).

**Table 5:** Cytogenetic, genetic, and transcriptional profiles of tumor DNA in samples from different regions (cfDNA, CMMCs, BM, or EM)

Samples	Methods	Observations	References
NDMM and RRMM CMMC vs. BMPC	FISH	CMMCs reflected cytogenetic changes in BM clonal PCs and consisted of unique cytogenetic subclones of BM clonal PCs	[62]
MM CMMC vs. BMPC	FISH	The status of 13q deletions was consistent with FISH results from paired BM clonal PCs	[42]
SMM and NDMM CMMC vs. BMPC	FISH	The status of t (4;14), t (14;16), and 17p deletion in CMMCs were consistent with BM FISH results in 88%, 94%, and 94% of NDMM patients, and in 91%, 90%, and 80% of SMM patients	[39]
MM CMMC vs. BMPC	Microarray CA and GEP	1. The concordance of CA between BM clonal PCs and CMMCs was 100% 2. Unsupervised clustering correctly clustered GEP of BM clonal PCs and CMMCs in 9 of 12 cases	[69]
NDMM CMMC vs. BMPC	WES Mutations and CNA	1. 90% of mutations in CMMCs were present in BM. 93% of mutations and 100% of clonal mutations in BM were present in CMMCs 2. The concordance of arm-level CNAs between BM clonal PCs and CMMCs was 92%	[92]
NDMM and RRMM CMMC vs. BMPC vs. EMPC	WES and microarray Mutations, CNA, and translocation	1. High concordance in the mutational profiles of three spatially distributed tumor samples at the individual level 2. 68% of mutations were shared by all three clones. CMMCs carried mutations in 92% of genes in BM or EM clonal cells 3. 82% of sSNV, 95% of arm-level sCNA, and only 39% of translocation in BM clonal PCs was present in CMMCs	[37]
MM (CMMC only)	scDNA-seq: CNA	CNA patterns were overall conserved with subclonal alterations at the individual level	[93]
MM (CMMC only)	scDNA-seq: CNA	CNA profiling revealed frequent convergent alterations at the individual level	[94]
MM CMMC vs. BMPC	scDNA-seq: CNA	1. CNA patterns in CMMCs were consistent with those in paired BM clonal PCs 2. The single-cell CNA of CMMCs was highly correlated with cytogenetics in the BM evaluated by karyotyping and FISH	[52]
MGUS, MM CMMC vs. BMPC	scDNA-seq: mutations scRNA-seq: translocation	1. 100% of targeted mutations (e.g., NRAS, KRAS, BRAF, IRF4, and TP53) in BM clonal PCs were confirmed in CMMCs 2. Some recurrent mutations were more abundant in CMMCs than those in BM clonal PCs 3. Translocations in CMMCs were confirmed by FISH in the BM	[51]
MM CMMC vs. BMPC	scRNA-seq	1. The gene expression signatures of CMMCs highly reproduced the transcriptional states in BM clonal PCs 2. There were a few differential expressions likely resulting from different environments (e.g., CRIP1 and KLF6)	[64]
MGUS, NDMM, and RRMM CMMC vs. BMPC	scRNA-seq Bulk RNA-seq Microarray	1. A significant correlation was observed in gene expression between CMMCs and BM clonal PCs 2. Genes involved in cytoskeleton reorganization and actin filament binding, migration/invasiveness, cellular adhesion, inflammation, coagulation, and cholesterol homeostasis were overexpressed in CMMCs. Genes involved in cell cycle were downregulated in CMMCs 3. Pathways involved in inflammation, angiogenesis, hypoxia, apoptosis, and epithelial–mesenchymal transition were upregulated in CMMCs. Pathways involved in cell cycle were downregulated in CMMCs	[65]
MM with EM CMMC vs. BMPC	scRNA-seq	1. CMMCs and BM clonal PCs tended to cluster in the same cell type 2. CXCL7 and secretion-related genes were significantly upregulated in CMMCs compared with those in BM clonal PCs	[35]
MM with EM CMMC vs. BMPC	scRNA-seq	S1PR2 was significantly upregulated in CMMCs compared with that in BM clonal PCs	[66]
NDMM, RRMM cfDNA vs. BM	LB-seq Mutations	1. 96% of mutations in BM clonal PCs were detected in paired cfDNA with high specificity (> 98%) 2. Mutant VAFs and the subclonal hierarchy of multiple mutations were highly concordant between cfDNA and BM	[3]
NDMM, RRMM cfDNA vs. BM	OMD Mutations	1. 24.2% of mutations (in KRAS, NRAS, BRAF, and TP53) detected in cfDNA were missed by a single-point BM biopsy 2. 38 of 97 mutations identified in BM clonal PCs were confirmed in matched cfDNA	[10]
NDMM, RRMM cfDNA vs. BM	OMD and TAS Mutations	1. More cfDNA-specific mutations (in KRAS, NRAS, BRAF, and TP53) were identified in RRMM cases than those in NDMM cases 2. The frequency of mutations in the DNA repair genes in cfDNA was significantly higher than those in BM clonal PCs, whereas the frequency of RAS–RAF pathway mutations was equivalent between cfDNA and BM clonal PCs	[78]
RRMM cfDNA vs. BM	OMD Mutations	1. A combination of cfDNA and BM clonal PCs detected more mutations (80%) than BM clonal PCs alone (60%) 2. 33%, 27%, and 40% of the total mutations were shared, BM-specific, and cfDNA-specific, respectively	[80]
MM cfDNA vs. BM	ddPCR Mutations	1. Mutations present in the BM clonal PCs was identified in cfDNA in 18 of 19 cases 2. 34/35 mutations present in the BM clonal PCs was identified in cfDNA	[75]
MM cfDNA vs. BM	ddPCR Mutations	1. The concordances of cfDNA and paired BM clonal PCs for KRAS Mx, NRAS Q61, and NRAS G12/G13 were all 100% 2. The concordance of cfDNA and paired BM clonal PCs for BRAF V600Mx was 76% 3. The positive rate of BRAF, KRAS, and NRAS mutations in the BM tumor cells (34%) was significantly lower than that in cfDNA (53%)	[79]
MGUS, SMM, NDMM, RRMM cfDNA vs. CMMC vs. BM	WES ULP-WGS Mutation and CNA	1. A strong correlation in the large CNAs was observed between matched cfDNA and CMMC and BMPCs 2. 99% of clonal mutations and 81% of CNAs in BM were identified in cfDNA and/or CMMCs. 83% of non-silent clonal mutations in BM were confirmed in cfDNA. 88% of non-silent clonal mutations in cfDNA were confirmed in BM. 96% of non-silent clonal mutations in cfDNA were confirmed in CMMCs, whereas 84% of non-silent clonal mutations in CMMCs were confirmed in cfDNA	[46]
NDMM, RRMM cfDNA vs. BM	LP-WGS and WES Mutation and CNA	1. Overall concordance of CNAs between cfDNA and BM was 90.5%. All mutations in driver genes were identified in both cfDNA and BM 2. 93% of clonal mutations in BM were confirmed in cfDNA. 91% of clonal mutations in cfDNA were confirmed in BM	[49]
NDMM, RRMM cfDNA vs. BM	ULP-WGS CNA	1. Overall concordance of CNAs between cfDNA and BM was 67%. 12% and 21% of CNAs were BM-specific and cfDNA-specific, respectively 2. The status of 1q21 gain and 17p13 deletion in cfDNA profiles were consistent with the results in BM clonal PCs in in 78% of cases	[48]
SMM and MM cfDNA vs. BM	ULP-WGS and NGS Mutations and CNA	1. Almost all the mutations identified in the BM clonal PCs were confirmed in cfDNA 2. The concordance of CNAs between cfDNA and BM clonal PCs was higher in MM cases (51%) than in MGUS and SMM cases (14%)	[56]
MGUS, SMM, MM cfDNA vs. BM	NGS Mutations	1. Recurrent genes (e.g., NRAS, KRAS, TP53, TRAF3, FAM46C, CYLD, DIS3, BRAF, and IRF4) were detected in cfDNA 2. 72% of mutations in BM were confirmed in cfDNA 3. cfDNA profiling detected 100% of mutations in the BM when VAF of mutations was ≥ 5% in BM	[57]
RRMM cfDNA vs. BM	NGS Mutations	1. The ratio between the SNV number in PC and in ctDNA was greater than 80% in more than half of the patients 2. Key driver gene mutations were exclusively detected in ctDNA in 48% of patients, which was likely to be missed in the BM	[76]
NDMM (case) cfDNA vs. BM	NGS Mutations and CA	1. t (11; 14) in BM clonal PCs was confirmed in cfDNA. Monosomy 13, which was suspicious positive in BM, was detected in cfDNA 2. The VAF of the mutation in KRAS was significantly lower in cfDNA than that in BM clonal PCs	[95]
MM with/without EM cfDNA vs. BM vs. EM	NGS Mutations	1. 66.67% and 31.25% of mutations in EM clonal PCs were detected in paired cfDNA and paired BM clonal PCs, respectively 2. Somatic mutation concordance was higher between cfDNA and EM clonal PCs (87.3%) than between BM and EM clonal PCs (62.1%)	[59]
NDMM cfDNA vs. BM	NGS Mutations, translocation	1. More than 50% of the mutated genes were shared between ctDNA and BM. Mutations with the highest VAF were shared 2. A positive correlation was observed in VAF between ctDNA and BM samples 3. The detection rate of translocation in ctDNA was consistent with the detection rate in BM	[73]

### Circulating myeloma cells

According to the conventional FISH method, the cytogenetic alterations between CMMCs and BM clonal PCs were substantially correlated [39, 42, 62]. In recent years, to identify the molecular alterations in CMMCs, high-throughput techniques (e.g., microarray, WES, and WGS) and single-cell sequencing were employed. A high concordance (approximately 92%–95%) of arm-level CNAs was observed between matched BM and PB tumor cells across paired samples by WES [37, 92]. Most paired BM clonal PCs and CMMCs had high-risk CNAs in MM, including 1q21 amplification and 13q deletion [37, 46, 52, 92, 94]. There is insufficient evidence for the feasibility of IGH translocation evaluation with CMMCs by high-throughput methods. A small sample study found that translocation, including t(11; 14) and t(6; 14), was shared by BM clonal PCs and paired CMMCs when comparing the IGH translocation in BM using FISH and related oncogene (CCND1 and CCND3) in CMMCs [51]. When PB and BM samples from the plasma of patients with leukemia were compared using single-cell RNA sequencing (scRNA-seq), the status of the IGH-WHSC1 gene fusion was frequently consistent in both samples, with more fusions being found in the BM than that in the PB [96]. However, the WES concordance for translocations between matched BM and PB tumor cells was only approximately 39%, which was likely because of the operating process’ random DNA fragmentation [37].

The matched tumor samples from separate compartments (BM, EM, and CMMC) had a high degree of concordance regarding the number, type, and protein effects of mutations [37]. CMMCs were observed to have the majority of mutations (92%–93%) and approximately all clonal mutations (CCF > 0.9) that were altered in BM or EM tumor cells [37, 46, 92]. The most recurrent and potentially driver mutations in genes (e.g., KRAS, NRAS, BRAF, and TP53) were shared by tumor cells from the BM, circulation, and EM [37, 46, 51, 67, 92].

However, the existence of mutation heterogeneity was observed in the tumor clones from different compartments. Generally, CMMCs had a higher frequency of somatic mutations than BM clonal PCs [51]. Some clones identified in CMMCs are not present at the BM or EM biopsy site or only present at the EM biopsy site although not at the BM biopsy site [37, 46, 51]. Private mutations had a significantly lower CCF than shared mutations [37, 92]. The discordance could be the consequence of a population of MM cells whose VAF was too low in the BM biopsy sample to be detected or a population that was not present at the BM biopsy site but rather only in a distant BM or EM site. These findings suggested that CMMC analysis may reveal other molecular alterations that single-point biopsies missed, although reflecting multiple tumor sites in the body.

Generally, the CMMC transcriptional signatures highly resembled the BM transcriptional states at single-cell and bulk levels in each patient [35, 64, 65]. A microarray-based study also found that the GEP signatures of BM clonal PCs could be appropriately reflected by CMMCs [69]. However, the discordance of gene expression was still observed between tumor cells from the different compartments, likely resulting from the different environments (e.g., expression of CRIP1 and KLF6) [64] or tumor cell aggressiveness and dissemination (detailed explanation in *Mechanisms explaining MM trafficking through PB disease dissemination*) [35, 65, 66].

### Circulating cell-free DNAs

Since CtDNA contains the dominant clones that are generated from numerous separate foci, its presence in the circulation may represent a comprehensive tumor genome. Overall, an average of approximately 83%–93% of clonal mutations discovered in BM clonal PCs were confirmed in cfDNA, and approximately 88%–91% of clonal mutations discovered in cfDNA were confirmed in BM clonal PCs [46, 49]. Most recurrently mutated genes in MM and pan-cancer mutations were shared by matched cfDNA and BM samples [3, 10, 46, 49, 57, 73, 75, 76, 79, 80]. Furthermore, identical subclonal hierarchies were observed in paired BM and plasma samples from patients with MM with ≥ 3 mutations or several mutations in the same gene [3]. In patients with MM, cfDNA and BM samples showed high concordance of CNAs (86.4%–90.5%) [49, 74], and most MM-related sCNAs (e.g., 1q gain and 13q deletion) were shared by two samples [46, 95]. The can profile from cfDNA produced a corresponding risk classification in 78% of patients with MM as the one obtained from BM clonal PCs based on 1q21 gain and 17p13 deletion [48]. A previous study used cfDNA from the circulation to identify IGH translocation. Detection rates of IGH translocation, which was identified in the BM by FISH, were similar in ctDNA (approximately 73.7%) and BM samples (approximately 78.9%) by NGS. Some IGH translocations missed by BM-FISH could also be identified in ctDNA by FISH [73]. Another study reported that CCND1 mutation was detected in cfDNA from a patient with MM with t(11; 14) in BM tumor cells. Monosomy 13 was reliably identified in cfDNA despite only being equivocally detectable in the BM compartment in this instance [95].

In general, cfDNA had higher VAF and detection rate of the mutations in driver genes than BM samples [78–80]. Additionally, cfDNA carried some unique mutations overlooked by a single-site BM biopsy, which were presumably from a distant BM or EM site [3, 10, 46, 56, 73, 76, 78, 80]. Matched cfDNA samples (66.67%) were observed to have more EM lesion-related mutations than matched BM samples (31.25%), indicating that cfDNA may be a superior alternative to BM samples when EM lesion biopsies are unavailable [59]. However, the molecular profile of tumor PCs in the BM was not frequently accurately replicated by ctDNA sequencing, and several BM-specific molecular alterations were observed [10, 56, 78, 80]. Compared with other shared mutations, the VAF of these BM-exclusive mutations was relatively low [3, 56, 57, 78]. One possible explanation for these missed mutations by cfDNA is that tumor-related mutations in cfDNA had significantly lower VAF than DNA from BM clonal PCs [56, 76]. A higher TF of tumor-related CNAs and VAF of tumor-related mutations in BM clonal PCs increased the likelihood of discovering tumor-specific mutations and CNAs in cfDNA [56, 57, 78]. Genetic analysis using both BM and plasma samples revealed more mutations (approximately 80%) than using BM samples alone (approximately 60%) [80].

#### Comparison between circulating myeloma cells and cell-free DNAs

To date, only one study systematically compared the molecule profiles of cfDNA and patients with CMMCs [46]. According to the study, CMMCs and cfDNA had high concordance in exome-wide somatic single-nucleotide variants and sCNAs. Overall, approximately 96% of non-silent clonal mutations found in cfDNA were confirmed in CMMC, whereas approximately 84% of non-silent clonal mutations found in CMMC were confirmed in cfDNA. They further proposed that both approaches provided distinct but complementary information. The combination of CMMCs and cfDNA detected almost all clonal mutations identified in the BM sample and uncovered other subclones that were missed in a single-site BM biopsy. TF evaluation in both CMMCs and cfDNA resulted in a higher proportion of patients who had at least one sample with sufficient tumor abundance for further deep sequencing (e.g., WES).

### Circulating cell-free RNAs

Circulating RNAs are generated via the following two main mechanisms: cell death and active secretion of vesicles containing RNAs [54]. According to a whole transcriptome study of extracellular RNA (exRNA) in the PB of MM patients and healthy controls, approximately 45% of the exRNA genes were protein-coding, and 85% of the identified genes were covered more than 70%, indicating that a sizable collection of gene transcripts was complete in the exRNA profile [97]. The researchers also discovered that the differentially expressed genes in the exRNA profile could be distinguished between MM patients and healthy controls. These findings suggested that exRNA profiles in the PB from MM patients could be potential biomarkers for MM detection and monitoring. The role of circulating non-coding RNA (primarily miRNA) in PB from MM patients have been comprehensively summarized by several reviews [98–100]. Here, we introduced the applications of cell-free messenger RNA (cf-mRNA) in PB in MM. With current MM therapeutics relying not only on direct anti-MM cell effects but also on immune cell response modulation, evaluating cfRNA could reflect a more comprehensive therapeutic response. Cf-mRNA analysis with a selected panel for MM noted that a high cf-mRNA level of CRBN and a low cf-mRNA level of IKZF1/3 at baseline were associated with a high risk of early disease progression [80]. According to another study, longitudinal cf-mRNA profiling of tumor-specific Ig rearrangement reflected the response to ASCT. Moreover, sequential monitoring of hematopoietic lineage-specific transcripts (e.g., erythrocytes and neutrophils) in cf-mRNA reflected hematopoietic reconstitution following ASCT and therapeutic response to stimulation with growth factors (e.g., EPO, G-CSF) [54]. A recent cf-mRNA global profiling in exosomes recapitulated the transition from MGUS to MM. This cf-mRNA panel, which contains a small number of genes (most of which have relatively high expression in the BM compared with other tissues and cell types), differentiated MM from premalignant conditions and healthy individuals [81]. These observations indicated that cf-mRNA may potentially provide a real-time approach to noninvasively evaluate BM function.

## Liquid biopsy of methylation biomarkers in cell-free DNAs

In addition to genetic information, cfDNA carries cancer-specific nongenetic information such as epigenetic information. One of the most frequent epigenetic alterations is aberrant DNA methylation. In recent years, many studies have revealed that detecting cfDNA methylation was a good approach for the screening and localization of cancer [101, 102]. The Circulating Cell-free Genome Atlas (CCGA), a population-based cancer screening program, is currently underway to develop a blood-based test for multi-cancer early detection, including MM [103]. The research found that methylation patterns evaluated by whole-genome bisulfite sequencing (WGBS) outperformed WGS and targeted sequencing in cancer detection and localization [104]. Across more than 50 cancer types, the false-positive rate of this methylation approach in cancer detection was less than 1% [103]. In a CCGA sub-study, the sensitivity of the WGBS was 73% in MM detection [104]. Furthermore, the methylation signature accurately predicted the origin of cfDNA in 92% ~ 100% of participants with plasma cell neoplasm [103, 105]. These observations suggested the potential value of cfDNA methylation profile in MM detection and monitoring, which still need to be confirmed by more studies. The cfDNA 5-hydroxymethylcytosine (5hmC) pattern could also be potential biomarkers for MM-related researches and clinical applications. Recently, a study that profiled genome‑wide 5hmC in circulating cfDNA from patients with NDMM and precursor states found that African Americans and European Americans had different 5hmC modifications, which correlated with their survival [106, 107].

## Disease monitoring

Sequential monitoring would help to early identify disease progression and recurrence before patients experience symptoms from overt relapse disease. Conventional monitoring, including PET-CT and single-site BM biopsy, cannot frequently perform in a timely manner, whereas serological markers are occasionally inadequate and nontrackable in some patients with MM. Given liquid sampling over multiple time points allowed the disease burden to be frequently tracked, liquid biopsy could be utilized as a dynamic tool to track tumor kinetics and define response or progression (Table 6).

**Table 6:** Longitudinal monitoring of therapeutic responses and disease status using liquid biopsy

Sample	Methods	Markers	Observations	References
CMMC	CellSearch	/	Patients who achieved remission had much lower CMMCs than at baseline, and who had relapsed had elevated CMMC levels	[39]
CMMC	IgH-qPCR	IgH rearrangement	In 66% of cases with progression, the 2IgH/b-actin ratio increased 4 months earlier than the relapse defined by the EBMT criteria	[5]
cfDNA	Ion Torrent	IgH rearrangement	A similar trend was observed between the levels of ctDNA and tumor dynamics evaluated using the IMWG criteria	[12]
cfDNA	NGS	IgK and IgL rearrangements	cfDNA profiles allowed for the detection of serologically measurable and unmeasurable MM (oligo-/non-secretory myeloma)	[36]
cfDNA	ASO-qPCR	IgH rearrangement	ctDNA levels decreased in response to therapy. The number of samples with undetectable ctDNA significantly increased over time	[8]
cf-mRNA	RNA-seq	IgH and IgL rearrangements	Longitudinal cf-mRNA profiling reflected the therapeutic response following melphalan-based treatment and ASCT	[54]
cfDNA	ULP-WGS	TF via CNA	The dynamics of TF in cfDNA were consistent with those of the FLC ratio in sequential monitoring	[46]
cfDNA	LP-WGS	TF via CNA	1. The dynamics of TF in cfDNA were consistent with those of sFLC in sequential monitoring 2. Sequential cfDNA analysis reflected the clonal evolution in BM clonal PCs when relapse and drug resistance occurred	[49]
cfDNA	ULP-WGS	TF via CNA	1. The kinetics of TF in cfDNA were consistent with those of BMPCs from SMM to MM to 3 months post-induction 2. The kinetics of TF in cfDNA were consistent with the changes in PET-CT	[74]
cfDNA	ULP-WGS	TF via CNA	1. A decline in cfDNA burden was observed as early as 1 week after treatment initiation 2. cfDNA showed robust and early detection of imminent relapse independent of low levels of serological parameters	[47]
CMMC	scDNA-seq	Somatic mutations	The clonal architecture of CMMCs exhibited remarkable similarities between remission and relapse	[51]
cfDNA	TAS	Allele fraction of mutations	The tumor fraction in cfDNA increased in the progression of SMM, which was consistent with the elevation in the FLC and BMPCs	[9]
cfDNA	NGS	Allele fraction of mutations	1. The ctDNA clonal structure was highly heterogeneous before and after six rounds of therapy 2. ctDNA samples from patients with CR and VGPR showed pathways enriched only in the clonal mutations, whereas ctDNA from patients with PR and PD showed pathways enriched only in the subclonal mutations	[73]
cfDNA	ddPCR	Allele fraction of mutations	1. High concordances were observed in ctDNA profiles among serial PB samples and between paired PB and BM samples 2. The serological response and the kinetics of the specific mutation in cfDNA showed discordance during the therapy 3. Clinical disease progression was associated with an increase in VAFs of NRAS and KRAS mutations	[3]
cfDNA	ddPCR	Allele fraction of mutations	The dynamics of the specific mutation in cfDNA showed similar or earlier disease detection than the serum light chain	[59]
cfDNA	WES and ddPCR	Allele fraction of mutations	1. Longitudinal sequencing of cfDNA reflected the clonal evolution during progression 2. In a patient with EM and oligosecretory MM, the VAF of NRAS Q61H in cfDNA continued increasing along with the persisting EM lesion, in contrast to the absence of FLC response, indicating the potential drug resistance of the clone	[91]
cfDNA (7 years)	ddPCR	Allele fraction of mutations	1. The kinetics of mutated VAF in cfDNA and M protein were highly covariant. ctDNA monitoring identified relapse parallel with or several months earlier than M protein and detected relapse in a case with light chain escape 2. Longitudinal sequencing of cfDNA reflected the change in genetic profile through the disease progress 3. In terminal disease, ctDNA reflected the development of disease better than M protein	[75]
cfDNA	ddPCR	Allele fraction of mutations	The dynamics of tumor-related mutations were concordant with the therapeutic response evaluated using paraprotein in serum, whereas cfDNAs were more sensitive for early detection of disease progression and relapse than sFLC	[108]
cfDNA	ddPCR	Allele fraction of mutations	1. The VAF of ctDNA coincided with or appeared to be better than the changes in sFLC in reflecting disease status and therapeutic response of patients with MM even in cases with light chain escape or nonsecretory MM 2. The longitudinal monitoring of cfDNA revealed the clones with differential therapeutic responses to different therapy	[10]
cfDNA	ddPCR	Allele fraction of mutations	1. Tumor fraction in cfDNA was correlated with changes in serum FLC or paraprotein and clinical progression in 87% of cases 2. Sequential sequencing revealed the clones with differential responses to drug treatment in individuals	[80]

### Disease monitoring using serologic assays versus liquid biopsy

Sequential serologic assays, including serum M protein, sFLC, and sIF, played significant roles in disease monitoring and response evaluation. In disease status evaluation at a single time point, concordance was observed between the CMMC level and serological measures in most studies [39, 41, 44–46]. CMMC assays represented as a more sensitive parameter than serological assays in some cases [44] and were detected in some cases who achieved CR [5, 23, 27, 36, 38, 39, 41]. For cfDNA assays, a good correlation was not frequently observed between the ctDNA level and serological measures [8–10, 36, 45–48]. A previous study showed that although the TF in cfDNA after treatment was concordant with IMWG responses in most patients, considerable variation in individual cfDNA TF was still observed, including several cases with very high TF despite apparent response according to the IMWG criteria [47]. However, it was also observed that a small group of cases with persistent M protein had complete clearance of CMMCs/cfDNA [5, 36, 38, 44, 45, 49, 75]. In sequential monitoring, the CMMC/ctDNA levels were generally concordant with tumor dynamics evaluated using the IMWG criteria (BMPCs, M protein levels, and sFLC ratios) [5, 10, 12, 46, 49, 59, 75, 80, 108]. However, in some studies, conventional serologic monitoring appeared insufficient and delayed for response assessment and progression and relapse prediction compared with early detection using liquid biopsy [5, 10, 47, 59, 75, 91, 108]. Therefore, liquid biopsy may complement the longitudinal evaluation of serologic parameters and help with the early detection of imminent progression/relapse, particularly in patients with serologically nontrackable diseases (e.g., LC escape, oligo-, or nonsecretory myeloma) [10, 36, 75, 91].

Possible causes of discordance between serologic assays and liquid biopsy may include the following: 1) M protein had a long half-life for days [109], whereas the half-life of cfDNA ranged from minutes to a few hours [110]. cfDNA may represent a prompt measure for the tracking of MM. 2) Serologic assays may be interfered with by therapeutic antibodies. 3) Serologic assays failed in evaluating disease status in patients with serologically nontrackable diseases, including nonsecretory MM [36]. 4) MM in the PB could be biased when the molecular properties were not involved in the detection panel. 5) The tumor cell burden in the PB was significantly lower than that in the BM (approximately 40–100 times lower) [5, 23, 41, 44]. Furthermore, cfDNA had significantly lower TF and VAF of tumor-related mutations than the BM [74]. MM in the PB could be missed when the disease burden did not reach the lower limit of the detection method.

### Response evaluation using the specific target of targeted treatment

The sequential analysis of target mutations in liquid samples could track the response to targeted therapies more frequently and comprehensively than BM biopsy. In a study of trametinib (a MEK inhibitor) in patients with MM with BRAF, NRAS, or KRAS mutations, researchers observed that the clinical disease progression was associated with an increase in VAFs of NRAS and KRAS mutations, indicating the involvement of a MAPK pathway-dependent mechanism in the resistance to trametinib. The authors further observed some inconstancies between serological response and the dynamics of the specific mutation in cfDNA during the therapy, indicating the existence of clones with differential responses to treatment [3]. Another study based on a cohort treated with lenalidomide and CC-486 (oral azacitidine) proposed that the cf-mRNA dynamics of CRBN, IKZF1, and IKZF3 could act as a biomarker of response to lenalidomide-based therapy. Low CRBN expression and high IKZF1 and IKZF3 expression in cf-mRNA at baseline could be indicative of patients more sensitive to lenalidomide. By comparing the expression level at baseline and on C1D5, increased IKZF1 expression was found to be an early marker of response to therapy [80].

### Tracking clonal evolution and identification of drug resistance

MM is a highly heterogeneous and dynamic disease. Liquid biopsy can be a noninvasive and dynamic method for capturing real-time genetic events, reevaluating disease risk over time, and identifying potentially targetable oncogenes for precision therapeutics.

Genomic and transcriptional heterogeneity exists among different individuals with MM [51, 64]. A high degree of heterogeneity in CMMC CNA profiles was observed among different patients with MM by single-cell DNA sequencing (scDNA-seq) [93]. The transcriptional profile of CMMCs between the two patients also substantially differed based on scRNA-seq [51]. Resistance to a specific drug could be the result of the presence of one or more driver mutations in oncogenes and/or tumor suppressors. Patients with relapsed/refractory MM had a significantly higher absolute number and frequency of plasma-exclusive mutations in cfDNA than those with NDMM [10, 78]. Moreover, patients with different IMWG responses showed different mutation patterns in ctDNA after treatment. Patients who achieved CR/VGPR had enriched pathways only in clonal mutations, whereas those who achieved PD/PR had enriched pathways only in subclonal mutations [73]. Liquid biopsy could identify potential genes involved in drug resistance and guide personalized therapy in MM by comparing the molecular profile of patients with differential therapeutic responses.

Approximately all patients with MM eventually acquired drug resistance. Clonal heterogeneity and constant clonal evolution of individuals with MM are likely explanations for the emergence of drug resistance. Longitudinal monitoring of BMMC and cfDNA showed that distinct populations carried different mutations and acquired new mutations through the progression, and the dominance of the population changed, with the more resistant clones possessing a growth advantage [10, 91, 111]. cfDNA analysis provided information on subclonal architecture in MM and constructed the evolution tree using relative fractions of mutations from the blood [3]. For example, the RAS–RAF pathway mutations appeared to be dominant in BM samples, whereas the DNA repair gene variants had a more predominant presence in cfDNA, indicating that the RAS mutations may be ancestral to the DNA repair gene mutations [78].

Serial cfDNA sequencing further monitored the dynamic changes in mutation fractions. The CNA profile and mutational hierarchy of cfDNA were persistently concordant with the profile of BM clonal PCs in serial samples, suggesting that cfDNA provides a good source for reflecting the sequential molecule characterization of BMPCs [3, 49]. Potential drivers and pathways (e.g., protein kinase A signaling and Wnt/β-catenin signaling) involved in disease progression and drug resistance in a specific individual were identified by comparing the genetic profile in ctDNA samples at different time points during disease progression and relapse [49, 91]. Furthermore, clonal outgrowth and subclone disappearance co-existent with disease progression identified different clones with different therapeutic responses and provided critical clues regarding therapy selection and the predominant driver mutation involved in resistance to therapy [10, 75, 80, 91].

Serial CMMC monitoring may reflect the clonal evolution of PCs developing anchorage independence and growth potential outside of the BM microenvironment under therapeutic and immunological pressure. For example, a sequential sequencing of CMMCs using scDNA-seq revealed that CNA patterns were overall conserved in individuals, along with subclonal divergent alterations and convergent lesions, indicating the co-existence of branched tumor evolution and convergent alternations [93, 94]. Determining genomic alterations during disease progression and detecting the emergence of a drug-resistant MM clone could be achieved by comparing the mutational profile of CMMCs at several time points [26]. An obvious similarity existed in the clonal architecture of CMMCs during remission and at relapse, suggesting that the treatment did not selectively kill particular resistant subclones and that these subclones could be the source of disease relapse [51].

## Conclusion and prospect

Liquid biopsy allows for the minimally invasive detection of disease burden and molecular alterations in MM, as well as repeated sampling for monitoring treatment response, drug resistance, and the appearance of potential molecular targets. The use of liquid biopsy would improve disease evaluation, particularly in patients with precursor diseases, EM diseases, or serologically nontrackable diseases. For further clinical transformation, approaches of liquid biopsy in MM should be standardized to establish consistency to compare clinical trial data. To date, the sensitivity of liquid biopsy in MRD evaluation remains lower than that of BM-based MRD assay in MM. Methods with high sensitivity of liquid biopsy for MRD evaluation need to be further explored. For example, a method called phased variant enrichment and detection sequencing has been confirmed with extremely high sensitivity and specificity in MRD detection in the PB in lymphoma, which allowed for ctDNA detection in the ppm range in samples [112]. In recent years, nongenetic features of cfDNAs, such as DNA epigenetics (primarily methylation), fragmentation, and topology, have broadened the utility of cfDNA [101]. So far, only a few studies have shown that cfDNA methylation can be used to detect MM. Further researches are required to exploring the cfDNA methylation pattern and other epigenetic patterns, and their utility in predicting therapeutic responses and disease prognosis in MM. In the future, the introduction of liquid biopsy into the disease evaluation of MM would provide a tool for the comprehensive and real-time assessment complementary to conventional methods, promoting the development of new risk stratification systems and individual therapy options.

## Data Availability

Not applicable.

## Abbreviations

ASCT: Autologous stem cell transplantation
ASO: Allele-specific oligonucleotide
β2-MG: Beta2-microglobulin
BM: Bone marrow
BMPC: Bone marrow plasma cell
CA: Chromosomal abnormality
CCF: Cancer cell fraction
CCGA: Circulating Cell-free Genome Atlas
CDC6: Cell division cycle 6
CENPF: Centromere protein F
cfDNA: Cell-free DNA
cf-mRNA: Cell-free messenger RNA
cf-NA: Cell-free nucleic acid
CMMC: Circulating multiple myeloma cell
CNA: Copy number aberration
CR: Complete response
CRIP1: Cysteine-rich intestinal protein 1
ctDNA: Cell-free tumor DNA
CXCL12: C-X-C motif chemokine ligand 12
CXCR4: C-X-C motif chemokine receptor 4
ddPCR: Droplet digital polymerase chain reaction
DS: Durie–Salmon
EM: Extramedullary
EMT: Epithelial–mesenchymal transition
EPO: Erythropoietin
exRNA: Extracellular RNA
FACS: Fluorescence-activated cell sorting
FISH: Fluorescence in situ hybridization
G-CSF: Granulocyte colony-stimulating factor
GEP: Gene expression profiling
Ig: Immunoglobulin
IGH: Immunoglobulin heavy chain
IMWG: International Myeloma Working Group
ISS: International Staging System
KLF6: Kruppel-like factor 6
LC: Light chain
LDH: Lactate dehydrogenase
MACS: Magnetic cell sorting
MAPK: Mitogen-activated protein kinase
MFC: Multicolor flow cytometry
MGUS: Monoclonal gammopathy of unknown significance
MRD: Minimal residual disease
MM: Multiple myeloma
M protein: Monoclonal protein
NDMM: Newly diagnosed multiple myeloma
NGF: Next-generation flow cytometry
NGS: Next-generation sequencing
PB: Peripheral blood
PC: Plasma cell
PCR: Polymerase chain reaction
OS: Overall survival
PET-CT: Positron emission tomography-computed tomography
PFS: Progression-free survival
PPV: Positive predictive value
PR: Partial response
qPCR: Quantitative polymerase chain reaction
R-ISS: Revised-International Staging System
Scr: Serum creatinine
scDNA-seq: Single-cell DNA sequencing
scRNA-seq: Single-cell RNA sequencing
SD: Stable disease
sFLC: Serum free light chain
sIF: Serum immunofixation electrophoresis
SMM: Smoldering multiple myeloma
S1RP2: Sphingosine 1-phosphate receptor 2
sSNV: Somatic single-nucleotide variant
TF: Tumor fraction
ULP-WGS: Ultra-low pass whole-genome sequencing
VAF: Variant allelic frequency
VGPR: Very good partial response
WES: Whole-exome sequencing
WGBS: Whole-genome bisulfite sequencing
WGS: Whole-genome sequencing
WHSC1: Wolf-Hirschhorn syndrome candidate gene-1
